# Simultaneously Enhanced Thermal Conductivity and Breakdown Performance of Micro/Nano-BN Co-Doped Epoxy Composites

**DOI:** 10.3390/ma14133521

**Published:** 2021-06-24

**Authors:** Chuang Zhang, Jiao Xiang, Shihang Wang, Zhimin Yan, Zhuolin Cheng, Hang Fu, Jianying Li

**Affiliations:** 1 State Key Laboratory of Electrical Insulation and Power Equipment, Xi’an Jiaotong University, Xi’an 710049, China; zhangchuang@stu.xjtu.edu.cn (C.Z.); wangshih@mail.xjtu.edu.cn (S.W.); yanzhimin@stu.xjtu.edu.cn (Z.Y.); czlxjt@stu.xjtu.edu.cn (Z.C.); fh291009595@stu.xjtu.edu.cn (H.F.); 2 Power China Hubei Electric Engineering Co., Ltd., Wuhan 430000, China; xiangtian886633@stu.xjtu.edu.cn

**Keywords:** bipolar square wave voltage, boron nitride, micro/nano co-doping, epoxy resin, thermal conductivity, breakdown

## Abstract

Micro/nano- BN co-doped epoxy composites were prepared and their thermal conductivity, breakdown strength at power frequency and voltage endurance time under high frequency bipolar square wave voltage were investigated. The thermal conductivity and breakdown performance were enhanced simultaneously in the composite with a loading concentration of 20 wt% BN at a micro/nano proportion of 95/5. The breakdown strength of 132 kV/mm at power frequency, the thermal conductivity of 0.81 W·m^−1^·K^−1^ and voltage endurance time of 166 s were obtained in the composites, which were approximately 28%, 286% and 349% higher than that of pristine epoxy resin. It is proposed that thermal conductive pathways are mainly constructed by micro-BN, leading to improved thermal conductivity and voltage endurance time. A model was introduced to illustrate the enhancement of the breakdown strength. The epoxy composites with high thermal conductivity and excellent breakdown performance could be feasible for insulating materials in high-frequency devices.

## 1. Introduction

Solid-state transformer (SST), an intelligent device for voltage grade conversion and power transmission, has been extensively investigated due to its superiority in efficiently transmitting clean energy by distributed generation and microgrid [1,2]. Casting dry-type SST with the merits of small size, excellent mechanical strength and great anti-corrosion performance properties, are widely used in medium voltage electrical networks [3,4]. Epoxy resins are the main insulation materials of casting dry-type transformers [5], owing to their good adhesion, great insulation performance, outstanding chemical stability and dielectric properties [6,7,8,9,10]. The epoxy materials in SST, different from traditional electrical devices, operate under a high-frequency high-voltage bipolar square wave. They have to endure a high electric field, as well as a high temperature generated by the high-frequency bipolar square wave [3,4,5,11,12,13]. Therefore, the epoxy composites with high thermal conductivity and excellent insulating ability under high frequency bipolar square wave voltage become the crucial issues for improving the reliability of the devices [5,14,15,16].

Numerous works about enhancing the thermal conductivity and breakdown strength of epoxy resin materials have been reported. Epoxy composites with micro- α-Al_2_O_3_ encapsulated by boron nitride (BN) nanosheets were reported by Huang et al. The thermal conductivity of 2.43 W·m^−1^·K^−1^ was obtained at 65 vol% filler loading [17]. Enhanced thermal conductivity of 0.85 W·m^−1^·K^−1^ at total filling content of 50 wt% was reported by Gao et al. when H-BN platelets and α-Al_2_O_3_ nanoparticles were loaded into an epoxy resin matrix [18]. An electric field was used by Du et al. to induce nanoparticle (BN & SiC) orientation in an epoxy resin matrix to obtain higher thermal conductivity, which improved the thermal conductivity to approximately 0.4 W·m^−1^·K^−1^ with a mass fraction of 40% [19]. Glass fiber cloth reinforced epoxy composite produced by Xiao et al. achieved improved thermal conductivity of 2.36 W·m^−1^·K^−1^ in-plane and 0.76 W·m^−1^·K^−1^ through-plane at a micro-h-BN content of 29.6 wt% [20]. However, the breakdown performance of the epoxy composites, which is the key parameter of insulating materials, was not much concerned in the works mentioned above. Wang et al. prepared epoxy composites with micro and nanofillers, which exhibited an increase of around three times in through-plane thermal conductivity but little enhancement of dielectric breakdown strength compared to pure epoxy [21]. Silicon carbide-boron nitride nanosheets were loaded into the epoxy matrix by Han et al. to obtain the thermal conductivity of 0.89 W·m^−1^·K^−1^ at a loading content of 20 wt%. Nonetheless, the breakdown strength of the epoxy composite was only 22.1 kV/mm [22]. 

Although the addition of micro fillers is beneficial to improving thermal conductivity, it inevitably increases the viscosity of the uncured mixtures and decreases breakdown strength. Reversely, despite the excellent breakdown strength of nanocomposites, their thermal conductivities are lower because of forming a large amount of interfacial area [23,24]. This raises the issue of whether the thermal conductivity and breakdown performance could be simultaneously enhanced by co-doping micro and nanofillers into the epoxy matrix. Thermally conductive paths can be formed by micro fillers to increase thermal conductivity. Meanwhile, proper nanofillers loading introduces deep traps to prevent the charges injection [25] and capture the mobile charges [26], which helps to improve breakdown performance [25]. Simultaneously improving thermal conductivity and breakdown performance of epoxy composites might be promising by combining the advantages of both micro and nanofillers.

In this work, micro and nano-BN fillers modified by KH550 were loaded into an epoxy resin matrix to prepare epoxy composites with simultaneously improved thermal conductivity and breakdown performance. Additionally, the effect of micro and nano-BN co-doping on thermal conductivity, breakdown strength at power frequency and voltage endurance capability under a bipolar square wave field with high frequency and high voltage of the epoxy composites were studied.

## 2. Sample Preparation and Experiments

### 2.1. Sample Preparation

**Figure 1:**
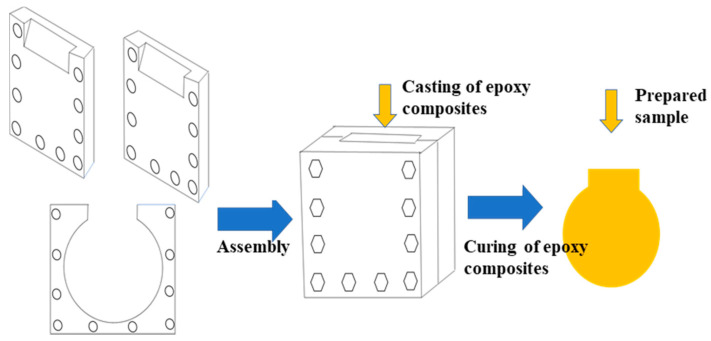
Diagram of the mold used for preparing the sample.

Surface modified micro (1~3 μm)- and nano (50 nm)- BN were loaded into the epoxy resin matrix, consisting of diglycidyl ether of bisphenol-A (E-51 type), methyl tetrahydrophthalic anhydride (MTHPA) and 2,4,6-tri (dimethylamine) phenol (DMP-30) with mass mixing ratio of 100:80:1. Firstly, the mixture consists of the epoxy resin matrix, micro and nanofillers were stirred by Thinky Mixer ARE-250 (THINKY, Tokyo, Japan) with rotation and revolution at 2200 rpm for 15 min. Then, the suspension was degassed at 2,000 rpm for 15 min [27,28,29]. The air bubbles were removed during the process of degassing. The uncured composite was then poured into a mold made of stainless steel. The mold consisted of three parts where two boards hold a clamping piece with a target size of thickness, just like a sandwich. The mixture was poured into the mold from the open port. A diagram of the mold is shown in Figure 1. Finally, the samples were obtained after being cured at 80 °C for 2 h, 105 °C for 2 h, and 120 °C for 4 h. Samples used for the breakdown performance test were 0.2 mm in thickness while those for the thermal conductivity test were 10 mm × 10 mm × 1 mm in size. 

Epoxy composite samples with varied loading contents of fillers and different micro/nano-BN proportions were fabricated. Pristine epoxy resin, epoxy/micro and epoxy/micro/nano-BN composites were labeled as EP, EPMx and EPMNx-y, respectively, where x stands for the total filler mass percentage while y represents the nano filler’s mass percentage of the total fillers. For example, EPMN20-5 means the mass percentage of all the fillers is 20 wt%, where nano-BN accounts for 5%.

### 2.2. Experiments

Scanning electron microscope (SEM) images were taken by VE-9800S (Keyence, Osaka, Japan). Mettler differential scanning calorimetry (DSC) 822e (Netzsch, Selb, Germany) was used to conduct the DSC test. The heating rate was set to 5 °C/min from 30 °C to 200 °C in nitrogen. Thermogravimetric analysis (TGA) was measured by Mettler TGA/SDTA 851 (Mettler Toledo, Zurich, Switzerland) in air. The heating rate was set to 20 °C/min from 50 °C to 650 °C.

A thermal conductivity test was performed by a thermal conductivity measurement instrument LFA447 (Netzsch, Selb, Germany) at room temperature where the laser flash method was applied. The dielectric properties were obtained by a broadband dielectric spectrometer (BDS) CONCEPT 80 (Novocontrol Technology, Frankfurt, Germany). For the BDS study, gold was evaporated on both sides of the slice samples with diameters of 22 mm and 25 mm, respectively. The tested temperature and frequency were range from 20 °C to 200 °C and from 10^−1^ Hz to 10^6^ Hz, respectively.

The breakdown strength and voltage endurance measurements were conducted by a breakdown testing system at power frequency and high frequency bipolar square wave voltage with 8 kV, 25 kHz at room temperature, respectively. Both experiments were performed in transformer oil to keep off surface flashover. The electrode system consisted of two hemisphere copper electrodes with a diameter of 20 mm. The voltage breakdown test was conducted with a boost rate of 1 kV/s. A total of 15 samples were tested for every experimental condition. The breakdown strength is calculated according to the formula

*E* = *U*/*d*, (1)

where *E* is the breakdown strength, *U* is the breakdown voltage, and *d* is the sample thickness. The performance of voltage endurance was characterized by voltage endurance time, defined as the withstanding time between the time the voltage applied on the high voltage electrode and the time the breakdown happened.

## 3. Results and Discussions

### 3.1. Effects of Micro/Nano-BN Co-Doping on Microstructure of Epoxy Composites

**Figure 2:**
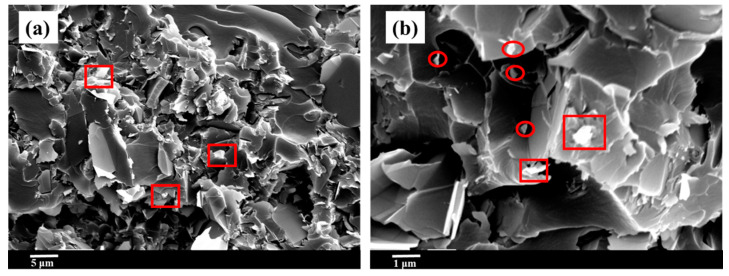
SEM images of (**a**) EPM20; (**b**) EPMN20-5, where white particles marked by rectangular, and dots, marked by circles, are the micro and nano-BN fillers.

Taking the EPM20 and EPMN20-5 as an example, cross-section SEM images of the epoxy composites with micro fillers and micro/nanofillers are shown in Figure 2. Those white particles, marked by rectangular, and dots, marked by circles, are the micro and nano-BN fillers, respectively. It is clear that the fillers were uniformly dispersed in the epoxy resin matrix. Moreover, the distribution of nanoparticles was more compact than the microparticles due to the small size of the nanoparticles.

**Figure 3:**
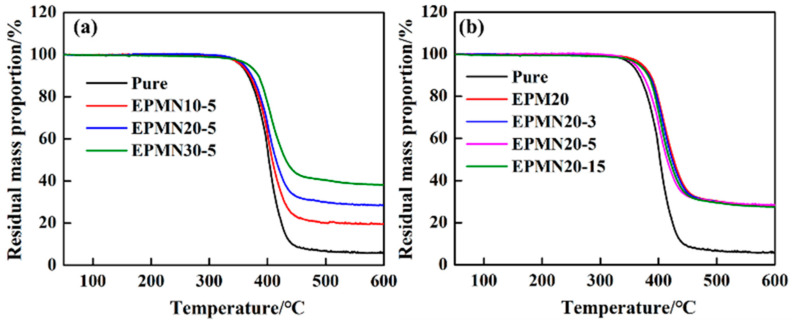
TGA curves of (**a**) EPMNx-5; (**b**) EPMN20-y.

Figure 3 shows the TGA curves of the epoxy resin matrix and epoxy composites, where the stability of the composites and residual mass proportion of BN fillers can be obtained. Figure 3a depicts the TG performance of the epoxy composites when the micro/nano-BN proportion was fixed. The residues, which mainly consisted of BN fillers, increased with the increase in loading content. Figure 3b illustrates the TG behavior of the epoxy composites when loading contents of BN were fixed. The residues of the composites are nearly constant due to the same loading contents of 20 wt%. As expected, the residual mass proportion was consistent with the original design of the experiment. The loading of fillers reduced the mass reduction rate of the composites, to some extent, improving the thermal resistance properties.

**Figure 4:**
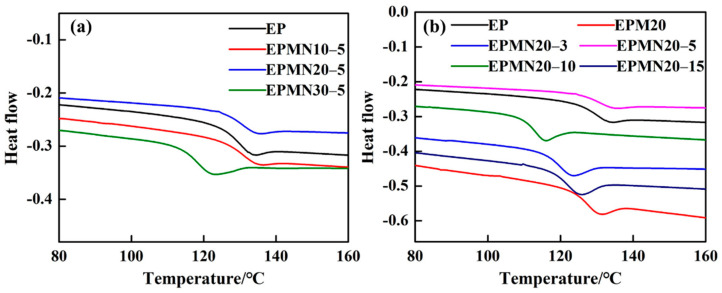
DSC curves of (**a**) EPMNx-5; (**b**) EPMN20-y.

The DSC curves of the samples are shown in Figure 4. The middle temperature was applied to indicate the glass transition temperature (*T*_g_) [8]. In those composites with a fixed proportion of micro/nano-BN, as shown in Figure 4a, *T*_g_ remained constant to about 127.9 °C when samples are slightly doped. A dramatic decrease in *T*_g_ appeared when the loading content reached up to 30 wt%. Figure 4b depicts *T*_g_ of the epoxy composites with different micro/nano-BN proportions at a loading content of 20 wt%, in which *T*_g_ were all lower than that of pristine EP, except EPMN20-5 and EPM20. The problem of interface compatibility between the fillers and the matrix is unavoidable, even though the surfaces of the fillers were modified. The particles, especially nano-BN, may introduce very small holes and defects because of the partly unavoidable aggregation of nano-particles [17,19], leading to the decrease in *T*_g_. However, micro BN doping shows no aggregation leading to moderate compatibility between the particle and the epoxy resin matrix. When the concentration of nano-particles is low, the tiny holes and defects have little impact on the crosslinking of the epoxy composites, and the *T*_g_ of the corresponding sample is slightly decreased. When the loading content of nano-BN is larger, the number of small defects increases quickly, and the curing process of the epoxy composites is significantly influenced, leading to the decrease in *T*_g_.

### 3.2. Effects of Micro/Nano-BN Co-Doping on Dielectric Properties of Epoxy Composites

**Figure 5:**
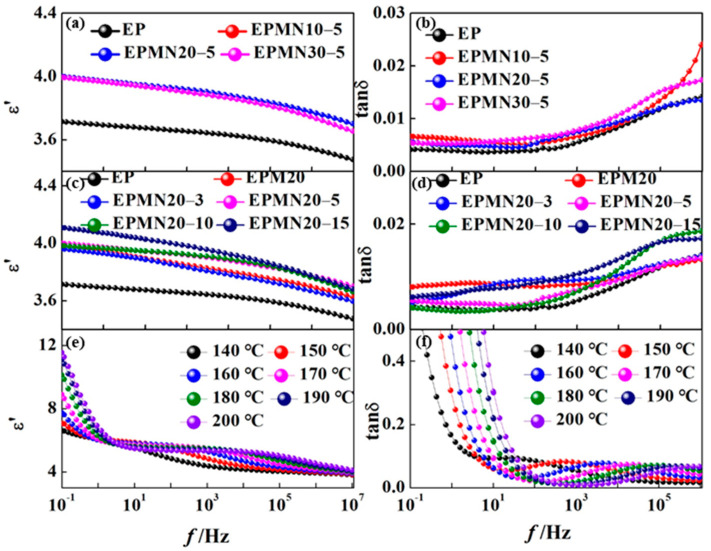
Broadband dielectric spectra of epoxy composites with (**a**,**c**,**e**) relative permittivity *ε′*; and (**b**,**d**,**f**) tan *δ*.

Figure 5 shows the dielectric properties of epoxy composites. The dielectric properties of materials are commonly characterized by complex permittivity ε*. It can be divided into two parts. One is the real part *ε′* representing the polarization ability of materials to respond to the applied electric field, including direct and alternating voltage. The other is imaginary part *ε″* relating to the dielectric loss during the process of response. The dielectric properties, namely dielectric constant and dielectric loss angle tangent, of pristine EP and BN/EP composites in the frequency range of 0.1 Hz~1 MHz and temperature range of 140~200 °C were investigated. Due to the requirement of high thermal conductivity but relatively low energy loss, the dielectric loss angle tangent tan *δ* = *ε″*/*ε′* should be focused on carefully.

Figure 5a,c show the relative permittivity *ε′* of the composite samples at room temperature. There are clear increments in *ε′* of EPMx and EPMNx-y compared with pristine EP in the studied frequency range. On the one hand, the *ε′* of loading fillers are larger than the epoxy resin matrix, leading to the increase in relative permittivity of composites [29]. On the other hand, the *ε′* of epoxy composites are found to be governed by the interfacial polarization determined by the number of orientable dipoles at the interface between the epoxy and the particles, which promote the relative permittivity of composites. The corresponding tan *δ* of the epoxy composites with varied loading contents of micro and nano-BN fillers are shown in Figure 5b,d. The tan *δ* with the value of 10^−2^ magnitude was obtained, in line with the dielectric losses reported in most papers [21,25,26,30,31,32]. The epoxy composites in SST have to endure high temperatures, therefore, the dielectric properties at different temperatures of EPMN 20-5 as an example were shown in Figure 5e,f. It is suggested that the *ε′* and peak frequency of tan *δ* increased with temperature increasing.

The power loss of dielectric originates from the electrical conduction and dielectric relaxation. When the frequency is low enough, the electrical conduction plays a prominent role in the dielectric loss, tanδ decreased with frequency increasing [33,34]. As the frequency increased, the effect of electrical conduction is reduced, which leads to a decrease in tan *δ*. Although the adding of micro or nanoparticles may lead to the variation of some values of electrical conductivity [35,36,37], the improvement of thermal conductivity and breakdown performance of the epoxy composites was focused on in this paper. The electrical conductivity will be considered carefully in our future work.

In general, tan *δ* decreases and increases with the increase in frequency in the low and high-frequency regions, respectively. The tan *δ* of epoxy composites are a little higher than that of pristine EP, increasing with the increment of loading contents of fillers. For example, the tan *δ* at 1 kHz of EP, EPMN10-5, EPMN20-5 and EPMN30-5 are 0.00563, 0.00674, 0.00759, and 0.00796, respectively. The tan *δ* of the EPMNx-5 exhibits little variation when the frequency is lower than 10 kHz, which may be attributed to the leading role of micro particles. However, the tan *δ* of the EPMNx-5 increased significantly than that of pristine EP when the frequency was higher than 10 kHz. The polarization of epoxy dipolar groups is more pronounced in the high-frequency region causing higher losses. As a result, the loss of pristine EP increased distinctly in this region. The side chains determining the *β*-relaxation peak are restricted in the vicinity of nanoparticles according to the multi-core mode [25], which leads to the reduction in loss of EPMN20-y composite materials when few nanoparticles are loaded into the EPM. The tan *δ* of EPM20, EPMN20-3, EPMN20-5, EPMN20-10 and EPMN20-15 are 0.00877, 0.00785, 0.00491, 0.00353 and 0.00764 at frequency of 5 Hz, which were reduced by 10%, 44%, 60% and 13%, respectively. The dielectric power loss of epoxy composites was increased by adding micro and nano-BN. The dielectric power loss of the sample can be expressed as:

(2) $$p=2\pi f\varepsilon_{0}\varepsilon_{r}E^{2}\tan\delta$$

Although the electromagnetic power used for measurements in dielectric spectra is too low for the increased temperature of the sample indicated in Figure 5, tan *δ* was not influenced by the testing voltage [33,34]. Due to high *f* and *E*, the sample may come to tremendous temperature rise when imposed high frequency and high voltage bipolar square wave field.

### 3.3. Effects of Micro/Nano-BN Co-Doping on Thermal Conductivity of Epoxy Composites

**Figure 6:**
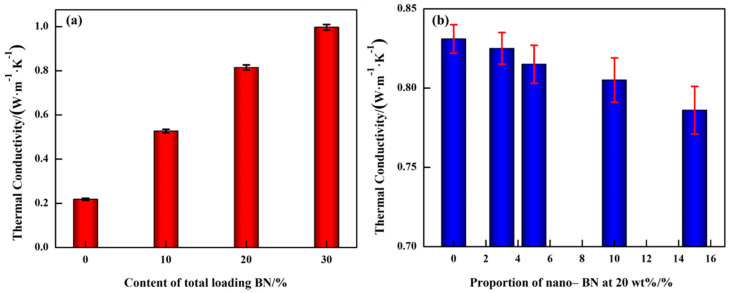
Thermal conductivity of epoxy composites with (**a**) fixed micro/nano-BN proportion; (**b**) fixed total loading content.

Figure 6 shows the thermal conductivity of the epoxy/BN composites at room temperature. The addition of BN could greatly improve the thermal conductivity compared with pristine EP of 0.21 W·m^−1^·K^−1^. When the micro/nano-BN proportion is fixed, the thermal conductivity increases with the increment of loading content, as illustrated in Figure 6a, especially when the loading content is higher than 10 wt%. The thermal conductivity of EPMN10-5, EPMN20-5 and EPMN30-5 are 0.53, 0.81 and 0.99 W·m^−1^·K^−1^, which are 152%, 286% and 371% higher than that of pristine EP, respectively. Tendency of the thermal conductivity, shown in Figure 6b, is EPM20 > EPMN20-3 > EPMN20-5 > EPMN20-10 > EPMN20-15 at the same concentration of 20 wt%. The thermal conductivity is lower when fewer nano-BN particles were added. It indicates that the nanofillers introduce greater interfacial thermal resistance than micro fillers because of the larger specific surface area [25]. The thermal conductivities of EPM20, EPMN20-3, EPMN20-5, EPMN20-10 and EPMN20-15 are 0.83, 0.82, 0.81, 0.80 and 0.79 W·m^−1^·K^−1^, which are 295%, 290%, 286%, 281% and 276% higher than that of EP, respectively. 

Theoretically, in order to enhance the thermal conductivity of composite materials, the longer thermally conductive paths or smaller thermal resistance originated from the disorder of crosslinking and filler/matrix interfaces were preferred [31]. Generally, thermally conductive paths can be maximized by improving the loading contents. Moreover, in order to build thermally conductive networks by improving the filling density, mixed fillers with different sizes and shapes are loaded into the epoxy resin matrix [32]. In this paper, the mixed fillers of micro/nano-BN are doped into an epoxy matrix. The thermal conductivity of composites reduced a little because of the thermal resistance introduced by nanoparticles.

**Figure 7:**
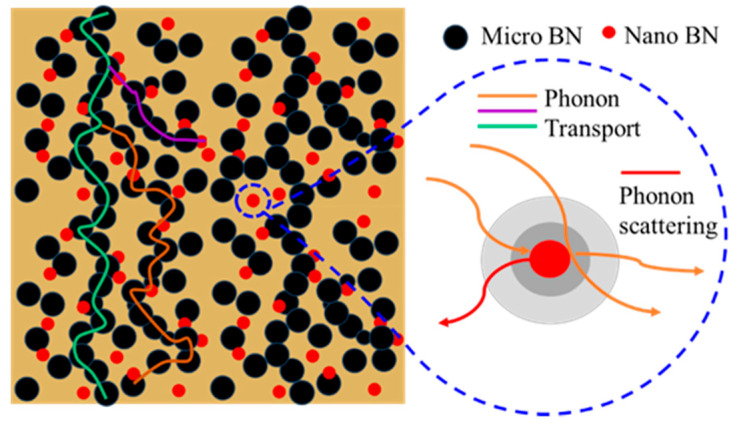
A schematic diagram of the combined effect of BN with different sizes on thermal conduction.

A schematic diagram of the synergistic effect of BN with different sizes on thermal conduction is shown in Figure 7, where the big black particles stand for the micro-BN, the minor red particles stand for nano-BN, and lines with colors of green, orange and blue represent the possible heat flows. The main thermally conductive paths are formed by closely contacted micro BN fillers to promote heat dissipation. The appropriate nano-BN substituted the micro BN with the same mass was loaded into the epoxy matrix to form interfacial traps, which may introduce thermal resistance. For the purpose of reducing the interface thermal resistance between the filler and epoxy resin matrix, both micro and nano-BN fillers were modified with KH550 to construct close interaction to transmit phonon efficiently between the filler and the matrix [32]. 

### 3.4. Effects of Micro/Nano-BN Co-Doping on Breakdown Performance of Epoxy Composites 

**Figure 8:**
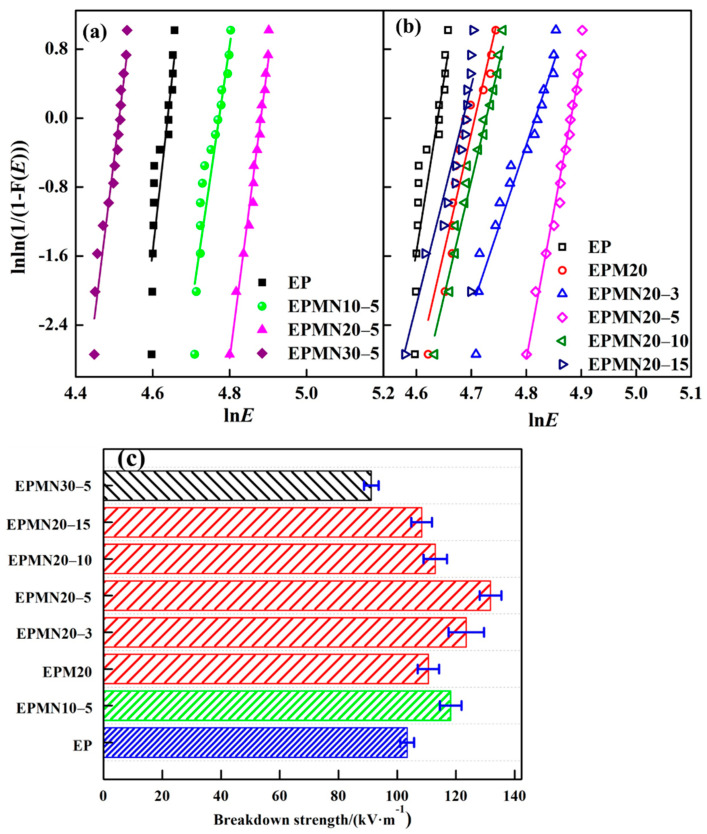
Breakdown performance at a power frequency of the epoxy composites with (**a**) Weibull distribution at fixed micro/nano-BN proportion; (**b**) Weibull distribution at fixed total loading contents; (**c**) breakdown strength.

**Figure 9:**
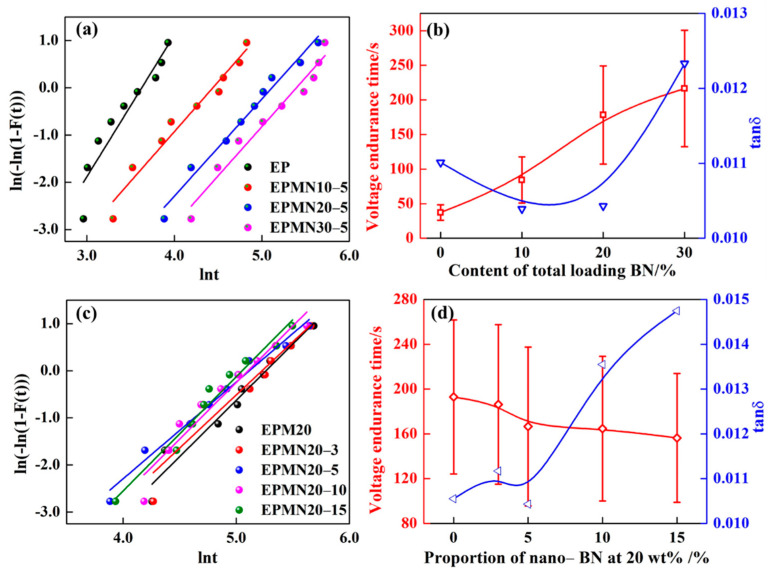
Voltage endurance time of the epoxy composites at (**a**), (**b**) fixed micro/nano-BN proportion; (**c**,**d**) at fixed total loading contents.

The breakdown performance of dielectric materials consists of breakdown strength and voltage endurance time, representing the short-time and long-time properties of materials serving as insulation, respectively. The breakdown strength at power frequency and voltage endurance time of EP, EPMx and EPMNx-y are shown in Figure 8 and Figure 9. In this paper, two-parameter Weibull distribution had been used to assess the breakdown performance of the epoxy composites according to IEEE 930 standard to assess the breakdown performance of the tested specimens, as treated by many works [38,39]. The relevant parameters of the distribution are the scale parameter representing the dielectric breakdown strength or the voltage endurance time at the cumulative failure probability (62.3%) and the shape parameter representing the level of data dispersion, higher values mean less scattering. In order to present the breakdown performance of the epoxy composite samples clearly, the shape parameters were removed.

It is observed from Figure 8a,b that the breakdown strength at power frequency for composites increases with filler loading less than 20%, regardless of micro loading or micro/nano co-doping. With an increase in the filler loading up to 30%, the breakdown strength at power frequency increases firstly and then decreases. It can be seen from Figure 8c that the breakdown strength of EP, EPMN10-5, EPMN20-5 and EPMN30-5 are 103, 118, 132 and 91 kV/mm, respectively. The adding of nano-particles substituting of the equal mass micro particles contributes to the breakdown strength at power frequency because of the special properties of nanofillers. For example, the breakdown strength of EPM20, EPMN20-3, EPMN20-5 EPMN20-10 and EPMN20-15 are 111, 123, 132, 113 and 108 kV/mm, respectively, increased by 8%, 19%, 28%, 10% and 5% comparing with EP, respectively. Loading microparticles or nanoparticles in a polymer matrix gives rise to increased dielectric strength because the fillers act as longer paths of a hindrance to breakdown compared with the pristine epoxy resin matrix. Furthermore, nanoparticles construct huge interfacial zone and tend to introduce more traps [6,7,8]. Once the inter-filler distances are small enough, the volume of the polymer matrix is reduced and the nanoparticles act like barriers to the movements of electrons between the electrodes [23,24]. 

The decreased breakdown strength of EPMNx-5(x > 20) can be associated with locally enhanced electric field strengths in polymer matrix closing to filler particles, because of the mismatch of permittivity. In addition, despite all the considerations taken during sample fabrication, defects (voids or weak cross-linking zone) may be introduced into the epoxy matrix by micro fillers [17,18,31]. The defects that appeared at the surface between filler and polymer matrix will cause additional field enhancement, leading to electrical discharges. The combination of the aforementioned effects will subsequently lead to an earlier breakdown of the composites at lower breakdown strengths than the pristine polymer.

Additionally, considering the fact that the epoxy composites operate under a high-frequency high-voltage bipolar square wave, voltage endurance time under high frequency bipolar square wave voltage is used to evaluate the dielectric strength of the composites (fixed at 25 kHz and 8 kV), as well. The experimental data were processed by Weibull distribution, as shown in Figure 9a,c, showing good linearity. The voltage endurance time of pristine epoxy resin is 37 s. Figure 9b,d depicts the tendency of voltage endurance time and dielectric loss angle tangent tanδ of the epoxy composites with varied loading contents. When the micro/nano- BN proportion of 95/5 is fixed, the voltage endurance time of the sample increases with the increment of concentration of fillers. The largest value of 220 s is obtained at loading contents of 30 wt%, which is 486% higher than that of pristine epoxy. When the loading contents of 20 wt% were fixed, the voltage endurance time performed as the tendency of EPM20 > EPMN20-3 > EPMN20-5 > EPMN20-10 > EPMN20-15. The corresponding voltage endurance time of 193, 186, 166, 164 and 156 s was obtained, increased by 422%, 403%, 349%, 343% and 322% than that of pristine epoxy, respectively. It indicates that the micro-BN plays a vital role in extending the voltage endurance time of epoxy composites under bipolar square wave filed with high frequency and high voltage.

Increasing tan *δ* may cause the temperature rise while improvement of thermal conductivity can inhibit temperature rise. Although there are two opposite effects, the voltage endurance ability of the composite under bipolar square wave voltage performed better than the pristine epoxy resin. Furthermore, the fact that voltage endurance time increases with the increment of the loading content of fillers suggests that the rising of thermal conductivity is more prominent than the increasing dielectric loss at 95/5 of the micro/nano-BN proportion with total filler concentration in the range of 10~30 wt%. The function of the two effects behaves distinctly with loading contents increasing.

The breakdown at bipolar wave voltage in this paper is a thermal breakdown because the voltage endurance time lasts for dozens of seconds. Heat is generated under high frequency and high electric field, leading to the increase in sample temperature. Moreover, the intensity of discharge in the voids introduced by the fillers was promoted, which improves the temperature of materials. If the heat generated is equal to that dissipated, the temperature is stable and a thermal breakdown is unable to take place. However, once the heat produced surpassed the heat dissipated, the temperature in the body of the sample rises continuously, causing the thermal breakdown of the sample eventually. According to ref. [40], the relation between the thermal breakdown electric field and various factors including heat-generating and dissipating was expressed as:

(3) $$E_{b}\;\propto \;\frac{\sqrt{\kappa }B}{\sqrt{f\varepsilon_{r}\tan\delta }}$$

where *κ* is the thermal conductivity, *ε*_r_ is the relative dielectric constant, tan *δ* is the dielectric loss angle tangent and f is the frequency of the electric field. It is clear that the electric field strength of thermal breakdown increases with the increment of thermal conductivity while it decreases with tan *δ* and frequency of the applied field. Similarly, the voltage endurance time is mainly affected by the dielectric loss and the thermal conductivity. Obviously, the voltage endurance time was improved by the thermal conductivity while decreased with dielectric loss. According to the experimental results, the thermal conductivity and the voltage endurance ability of epoxy composites under a high frequency bipolar square wave field were improved prominently. Meanwhile, the breakdown strength of the epoxy composites was enhanced.

Consequently, the results mentioned above revealed that the proper filling of nano-BN may improve the breakdown performance of epoxy composites at power frequency conditions, while the thermal conductivity should be considered prominently when used as insulating materials under high frequency bipolar square wave voltage. This paper may provide a direction to develop potential epoxy composites used in different conditions. Due to the limited experimental conditions, just 8 kV and 25 kHz in this paper, the limit of the usefulness of the composite under certain conditions will be considered in our future work.

## 4. Conclusions

In this paper, simultaneously enhanced thermal conductivity and breakdown performance were obtained in the epoxy composite with a micro/nano-BN proportion of 95/5 at a total loading concentration of 20 wt%. The breakdown strength at power frequency, thermal conductivity and voltage endurance time was 132 kV/mm, 0.81 W·m^−1^·K^−1^ and 166 s, respectively, which were approximately 28%, 286% and 349% higher than that of pristine epoxy resin. The prepared epoxy composite may serve as the insulating material applied in high frequency and high voltage devices.

## Data Availability

Not applicable.
